# Supplementary material for: Incidence of non-Hodgkin’s lymphoma among adults in Sardinia, Italy
Source: PLoS One. 2022 Feb 2;17(2):e0260078. doi: 10.1371/journal.pone.0260078 (PMC8809576; doi:10.1371/journal.pone.0260078)
Supplement: Attachment — Submitted filename: Response to reviewers.docx [file pone.0260078.s002.docx]

PLoS One Manuscript # PONE-D-21-08974

“*Incidence of non-Hodgkin’s lymphoma among adults in Sardinia, Italy”*

Response to the Editor

1. “…*ensure that your manuscript meets PLOS ONE's style requirements, including those for file naming*”

Title page, main text, and file names do now comply with PLOS ONE’s style requirements.

2. “*data from this study …available upon request*”

We took note of the Journal’s requirement for data availability, and we uploaded the file with the aggregated data as a supplementary file in submitting the revised version.

3. “*Figure 2 … contains map images which may be copyrighted”*

We contacted the person who created the map of the administrative borders of the Italian communes and made it available on the web site https:\\umap.geonue.com/en/map/confini-e-dati-statistici-dei-comuni-ditalia _297#8/40.102/8.973. As reported in the screenshot of the web page we accessed, the map was based on a software created by the National Association of the Italian Communes (ANCITEL) under an Open Database License (ODbL). As reported in the web site https:// opendatacommons.org/licenses/odbl/1-0/index.html, the ODbL license is a license agreement that allows users to freely share, modify, and use under the understanding that the resulting product will also made freely available to the public, and with the condition that the attribution of the original work would be acknowledged to the authors, the product would be shared alike, and the database would be kept open. Such conditions seem to us compatible with the CC BY 4.0 license. Therefore, in the revised version, we added a sentence acknowledging the authors of the map and the creator of the software used for the purpose under the ANCITEL ODbL license. We also uploaded the screenshot of the web page we accessed (in Italian), along with its translation to English, as an “other” file with the submission of the revised manuscript.

4. The “… *ethics statement should only appear in the Methods section* …”

The ethics statement is now the last paragraph of the Methods section.

5. “…*remove your figures from within …[the]… manuscript file, leaving only the individual TIFF/EPS image files, uploaded separately*.”

We removed Fig 2 from the manuscript file, and uploaded separately its parts as individual TIFF image files.

Response to the reviewers’ comments

Reviewer 1. Grammatical errors.

We are grateful to the reviewer for picking some grammatical errors in the text. We amended those and a few others. However, the plural form of the verbs “to confirm” (line 249) and “to indicate” (line 250) is correct as the subject (Cancer Registry data) is plural.

Reviewer 2.

1. “…*diagnostic biases caused by the important changes made to the classification of lymphomas during the last 30 years***”.**

We dedicated an entire paragraph, the first under the subheading “*Limitations*” from line 309 to 328 on page 10 to discuss this point. As we explain in the text, having the first author, himself a senior hematologist, collecting and revising each diagnosis of the incident hematological malignancies in 1974-2003 should reassure us and the readers about diagnostic bias due to the changes in the lymphoma classification that occurred along the study period.

2. ”…*the analysis of lymphoma as a whole* …[is]*… a second major concern that severely limits the transferability of these data to the clinic. Lymphomas are currently seen as a group of separate entities and every effort should be done to refer data also from cancer based registries to single entities*”. “*Are there any lymphoma specific data available at least for the most recent diagnoses, to try to identify if some lymphoma subtype was the cause of different geographical distribution?*”.

We definitely agree with the reviewer that every effort should be addressed to explore individual subtypes. However, the point is that the epidemiological analysis of time trends requires using a uniform definition of the disease entity across the time span object of the investigation. This imposes keeping using the initial definition. For the same reason, the IARC CI5 volumes and the Cancer Registries also keep using the NHL definition. A few studies have explored time trends, although within a still limited time frame (Morton L et al. Blood 2006;107(1):265-276; Luminari S et al. Hematol Oncol 2007;25:189-197; van Leeuwen MT et al. Int J Cancer 2014;135(9):2146-2156; Lim RB et al. Int J Cancer. 2015;137(11):2674-2687) or survival from (Al-Hamadani M et al. Am J Hematol 2015;90: 790–795; Ye X et al. BMJ Open 2017;7(7):e015106), major B-cell lymphoma subtypes. To the best of our knowledge, enough information might be currently available to explore the spatial distribution of lymphoma subtypes as well. We hope that for the future IARC would promote the registration of lymphoma subtypes besides keeping the old NHL definition, so to make possible what the reviewer proposes at a larger scale.

2. “…*possible temporal changes in the epidemiology of lymphomas during the study period. it would be interesting to analyze this trend separating the study frame in 2 or 3 periods and if number are not too small to identify and explain possible changes.”*

We are grateful to the reviewer for raising this interesting comment. To identify any joinpoint in the regression of NHL incidence against time, we plotted the residuals against the values predicted by the regression equation. As shown in figure 1, high or low residuals did not cluster in a specific range of predicted values. Therefore, we interpreted the upward trend we observed as continuous, with no evidence of a sudden change in slope, which might have been related to introduction of new diagnostic tests, or upgrade or new opening of onco-haematology facilities, or any acute environmental event. We added a new sentence at the end of the Time trend in NHL incidence paragraph of the Results section.

Figure 1. Residuals *vs* values predicted by the regression equation of 1974-2003 NHL incidence in Sardinia.

4. *“… compare these epidemiological data (i.e. world standardized incidence) with other available from other cancer registries in Italy and to discuss about the comparison (i.e. Luminari et al. Hematol Oncol 2007).”*

We again grateful to the reviewer for its useful suggestion. We retrieved the paper by Luminari et al., we extracted the corresponding figures from our data set, and we compared the age standardized incidence rates (World standard population) in males and females for the corresponding period. Rates from the Modena Cancer Registry were substantially higher in both genders. In the revised version, we added a paragraph under the subheading “Time trend in NHL incidence” to present and comment about such comparison.
