# Supplementary material for: Incidence of non-Hodgkin’s lymphoma among adults in Sardinia, Italy
Source: PLoS One. 2022 Feb 2;17(2):e0260078. doi: 10.1371/journal.pone.0260078 (PMC8809576; doi:10.1371/journal.pone.0260078)
Supplement: Attachment — Submitted filename: Broccia G etalR2 - Response to reviewers.docx [file pone.0260078.s003.docx]

PLoS One Manuscript # PONE-D-21-08974R2

“*Incidence of non-Hodgkin’s lymphoma among adults in Sardinia, Italy”*

Response to the reviewers’ comments

Reviewer 1.

1. Grammatical error (“*38th page - suggests not suggest*”).

The plural form of the present tense of the verb “to suggest” is used twice: one in the abstract (line 10) and one on page 10 of the manuscript (last line). In both instances, the plural form is correct as the subject (Cancer Registry data) is plural.

2. “*42 page* *- the communes associated with posterior probability are located on the extreme right of the incidence distribution curve of the 356 communes in the region would be better way to phrase and write than the current original one*”

We rephrased the statements that describe the location of high incidence communes in the graph and in the map as it follows: “*Another 22 communes are located on the extreme right of the incidence distribution curve of the 356 communes in the region, with a posterior probability of an excess NHL incidence in the total population ranging 80 – 94%. On the map, these communes tended to concentrate in the northern (20/35 communes) and central (12/35) areas of the island, with only three in southern Sardinia, including the major urban area of Cagliari*.” We are grateful for the reviewer suggestion, and we hope the sentence sounds better.
